# Feasibility and Safety of Low-Dose Mesenchymal Stem Cell Infusion in Lung Transplant Recipients

**DOI:** 10.1093/stcltm/szac051

**Published:** 2022-07-26

**Authors:** David Brett Erasmus, Nisha Durand, Francisco A Alvarez, Tathagat Narula, David O Hodge, Abba C Zubair

**Affiliations:** Mayo Clinic in Florida, Jacksonville, FL, USA; Mayo Clinic in Florida, Jacksonville, FL, USA; Mayo Clinic in Florida, Jacksonville, FL, USA; Mayo Clinic in Florida, Jacksonville, FL, USA; Mayo Clinic in Florida, Jacksonville, FL, USA; Mayo Clinic in Florida, Jacksonville, FL, USA

**Keywords:** lung, mesenchymal stem cells, Th1/Th2, transplantation, adult human bone marrow

## Abstract

**Background:**

We have previously shown bone marrow-derived mesenchymal stem cells (MSCs) may shift immune responses toward anti-inflammatory pathways and stabilize the course of obstructive chronic lung allograft syndrome (o-CLAD) after lung transplantation. In this study, we measured the response of lower dose infusions.

**Methods:**

We infused low-dose MSCs intravenously in 13 patients who had developed moderate-to-severe o-CLAD. Three had previously received an infusion of MSCs from a different donor and were re-dosed at 1 × 10^6^ MSC/kg, while 5 received a first dose at 1 × 10^6^ MSC/kg and five received an even lower dose at 0.5 × 10^6^ MSC/kg. We recorded pulmonary function tests before and after infusion, and patients were followed clinically for 12 months.

**Results:**

Infusions were well tolerated, and no significant adverse events were recorded in the first 30 days. There was significant decline (mean ± SD) in forced vital capacity (FVC) (3.49 ± 1.03 vs 3.18 ± 0.94 L, *P* = .03) and forced expiratory volume in 1 second (FEV1) (2.28 ± 0.86 vs 1.77 ± 0.49 L, *P* = .04) over the year preceding infusion. FVC (3.18 ± 0.94 vs 3.46 ± 0.99 L, *P* = .53) and FEV1 was not significantly changed (1.77 ± 0.49 vs 1.88 ± 0.75, *P* = .72) when comparing values immediately prior to infusion to those obtained 1 year after infusion, indicating a possible stabilizing effect on lung function decline due to o-CLAD.

**Conclusion:**

Intravenous infusions of bone marrow-derived MSCs are well tolerated in lung transplant recipients with moderate-to-severe CLAD. Low-dose MSCs appear to slow progression of CLAD in some patients.

## Graphical Abstract

**Figure:**
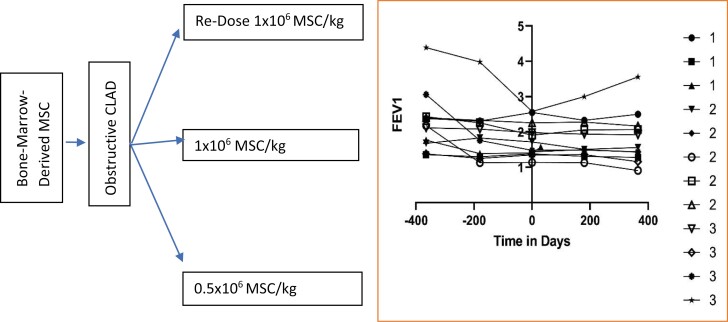
Forced expiratory volume in 1 second (FEV1 in liters) was measured before and after low-dose mesenchymal stem cell infusions (0.5 or 1 million cells/kg on day 0) in lung transplant recipients with moderate to severe chronic lung allograft dysfunction. Infusions were well tolerated and showed a stabilizing effect on FEV1 decline when measured 1 year after infusion (*P* = .72)

**Lessons Learned**

- Bone marrow-derived mesenchymal stem cells may be safely administered to lung transplant recipients with chronic rejection (CLAD).
- Preliminary results suggest this intervention may slow the decline in lung function associated with CLAD.

**Significance Statement**

Chronic rejection is the major cause of death and debility in lung transplant recipients who survive the first year. While survival after lung transplant has significantly improved over the past 2 decades, chronic rejection continues to be a problem. Any intervention that slows or stops the process will have a significant impact on survival. Preliminary results suggest mesenchymal stem cells should be investigated as a possible solution and they have shown some beneficial effects in other inflammatory lung diseases. We have shown that these cells can be safely administered to lung transplant recipients.

## Introduction

Lung transplantation offers prospects for better longevity and quality of life for a variety of end-stage lung diseases unresponsive to medical or surgical interventions.^1^ Despite improvements over the last decade, chronic lung allograft failure (CLAD) still limits long-term survival. In a retrospective cohort study of primary lung transplant recipients (1994-2011) reported to the International Society of Heart and Lung Transplantation Thoracic Transplant Registry, 79 896 person-years of follow-up showed that median bronchiolitis obliterans syndrome (BOS) (obstructive CLAD) free survival to be 3.16 (95% CI, 2.99-3.3) and 3.58 (95% CI, 3.53-3.72) years for single vs bilateral lung transplant recipients, respectively. Almost 90% of single and bilateral lung transplant recipients achieved the composite outcome of BOS (obstructive CLAD) or death 10 years after transplantation.^2^ Chronic rejection remains the most important factor limiting survival after lung transplantation. Several clinical phenotypes for CLAD have been identified, predominantly obstructive (BOS) or restrictive (restrictive allograft syndrome).^3–5^ All phenotypes are associated with a sustained decline in airflow, measured most reliably by a decline in forced expiratory volume in 1 second (FEV1) compared to baseline (average of 2 best FEV1 values post-transplant, measured at least 3 weeks apart), in the absence of acute rejection, airway stenosis or active infection. Several factors may predispose patients to develop CLAD. Repeated bacterial, fungal, or viral infection, episodes of acute rejection (both cell-mediated and humoral), gastroesophageal reflux disease (GERD) with aspiration, vascular injury, and others have been implicated.^6–9^ Medical therapy with azithromycin^10^ or montelukast,^11^ enhanced immunosuppression,^12, 13^ extracorporeal photopheresis, or medical and surgical intervention for GERD^14^ may stabilize some patients, but in others progressive decline is relentless. Re-transplantation as a last resort carries a worse prognosis than primary lung transplantation, and few patients qualify for re-transplantation.^15, 16^ Better interventions are required if lung transplantation is to no longer lag other solid organs for long-term survival. The development of novel and well-tolerated therapies for CLAD remains a high priority.

Mesenchymal stem cells (MSCs) are preferentially trapped in the lung after intravenous infusion, particularly in a setting of acute inflammation.^17, 18^ They are known to modulate the cellular immune system by suppressing effector T cells. They shift T-helper (Th) 1 to a Th2 immune response, thereby shifting immune responses toward an anti-inflammatory and tolerogenic phenotype.^19, 20^ They also exert an effect on B cells, thereby reducing antibody production.^20^ Autologous and allogeneic MSCs have been evaluated in the treatment of other inflammatory lung conditions, such as graft versus host disease (GVHD).^21^ In a prior phase I study at our institution (referred to in this study as phase Ia), we administered a single dose of MSCs to 9 patients, divided into 3 groups according to dose (4 × 10^6^, 2 × 10^6^, and 1 × 10^6^ cells/kg), with established moderate CLAD (6.6 ± 3.1-year post-transplant). All patients had an obstructive phenotype. Clinically, there was no discernible difference in gas exchange at 1 year and renal function was unaffected. Spirometry suggested a pattern of stabilization in FEV1 and forced vital capacity (FVC) over the year following infusion. The most beneficial biological effect of an increase in tolerance inducing Th2 cytokines and reduction in Th1 pro-inflammatory cytokines (IL-1-α, IL-6, IL-8) and chemokines (MIP-1α, MIP-1β) appeared to occur in the group receiving the lowest dose of MSCs.^22^ Others have also demonstrated that intravenous administration of bone marrow-derived allogeneic MSC is well tolerated in lung transplant recipients with moderate to severe CLAD and may provide stabilization to lung function.^23^

In this study, (referred to as phase Ib), we measured lung function and clinical parameters in lung transplant recipients with established moderate to severe CLAD and an obstructive phenotype after a single low-dose infusion of MSCs. We also re-dosed a small number of patients (*n* = 3) who had received MSCs in the previous (phase Ia) study. All patients received a single-dose intravenous infusion of bone marrow-derived mesenchymal stem cells (BMSCs).

## Materials and Methods

### Patient Population/Trial Design

The study patients were recruited from the clinical practice of the lung transplant program at Mayo Clinic in Jacksonville, Florida. This study was conducted under IND15807 from the US Food and Drug Administration (FDA), approved by the Mayo Clinic Institutional Review Board (Protocol #14-000025), and registered at ClinicalTrials.gov NCT02181712. Informed consent was obtained from all study participants.

Patients diagnosed with moderate to severe obstructive CLAD (Table 1), refractory to standard interventions were enrolled in this phase I study. Thirteen patients received MSC infusion, but one withdrew before the 60-day follow-up, after being deemed suitable for re-transplantation. Twelve patients (11 male and 1 female) completed follow-up. All subjects were enrolled between July 23, 2018 and September 18, 2020 and were followed for 12 months. Three patients who had participated in the phase Ia study received a second dose of MSCs in this phase Ib study (group 1). Demographic characteristics are summarized in Table 1. Thirteen patients (11 male and 2 female) were recipients of either bilateral (*n* = 10) or single (*n* = 3) lung transplants. Underlying pre-transplant diagnoses included idiopathic pulmonary fibrosis (*n* = 8), fibrotic NSIP (*n* = 1), idiopathic bronchiectasis (*n* = 1), primary ciliary dyskinesia (*n* = 1), cystic fibrosis (*n* = 1), and alpha-1 antitrypsin deficiency with severe emphysema (*n* = 1). Patients were generally of advanced age (mean 64.4 ± 11.2 years). The day of infusion was considered day 0. Baseline immunosuppression agents are included in Table 1. All patients were immunosuppressed by a calcineurin inhibitor and prednisone. All but one patient in group 1 and one in group 2 were also on a cell cycle inhibitor (mycophenolate or azathioprine). Baseline immune function was measured as follows: mononuclear cells (MNCs) were isolated from the peripheral blood collected at baseline and days 1 and 7 ± 1 using Ficoll-Paque PREMIUM 1.077 (Cytiva, Marlborough, MA). Isolated MNCs were evaluated for B cells (CD45^+^, CD19^+^), natural killer (NK) cells (CD45^+^, CD56^+^), and regulatory T cells (Tregs) (CD4^+^, CD25^+^) (Abs from Beckton Dickinson, Franklin Lakes, NJ). Data were acquired using an Accuri C6 Cytometer and analyzed using FCS Express. None of the patients had received a monoclonal antibody infusion or GCSF (granulocyte colony-stimulating factor) within the year preceding MSC infusion. Baseline PRA (pre-formed antibody) data are included in Table 2. Two patients had detectable PRAs, but none were donor-specific.

**Table 1.** Demographic characteristics of the 3 study groups.

Dosing	Age	Sex	Pre-Tx diagnosis	IS	Type Tx	cPRA %	Re-dose MSC	FEV1 % decline at infusion	Interval from prior MSC dose (years)	Time: Tx to MSC infusion (years)
Group 1 1 × 10^6^ MSC/kg	74	M	PCD	CyA, MMF, Pred	Bilateral	0	Yes	49	3.05	10.3
	73	M	IPF	Tac, AZA, Pred	Single	0	Yes	48	3.68	13.1
	64	M	IPF	Tac, MMF, Pred	Bilateral	0	Yes	52	3.26	8.45
Group 2 0.5 × 10^6^ MSC/kg	71	M	IPF	Tac, Pred	Bilateral	0	No	42	NA	6
	36	M	CF	Tac, MMF, Pred	Bilateral	4	No	53	NA	6.5
	65	F	IPF	Tac, MMF, Pred	Bilateral	0	No	49	NA	3.12
	72	M	IPF	Tac, MMF, Pred	Bilateral	32	No	45	NA	5.02
	70	M	IPF	CyA, Pred	Bilateral	0	No	32	NA	8.75
Group 3 1 × 10^6^ MSC/kg	72	M	BE	Tac, MMF, Pred	Bilateral	0	No	46	NA	9.5
	71	M	IPF	Tac, Pred	Single	0	No	32	NA	12.75
	62	M	IPF	Tac, MMF, Pred	Bilateral	0	No	48	NA	8.04
	48	M	α-1 AT	Tac, MMF, Pred	Bilateral	0	No	38	NA	2.47
	59^a^	F	f-NSIP	Tac, MMF, Pred	Single	0	No	53	NA	4.79
Mean ± SD	64.4 ± 11.2							45 ± 7		7.6 ± 3.4

Abbreviations: AZA, azathioprine; BE, bronchiectasis; CF, cystic fibrosis; CyA, cyclosporine; F, female; FEV1, forced expiratory volume in 1 second; f-NSIP, fibrosing nonspecific interstitial pneumonia; IPF, idiopathic pulmonary fibrosis; IS, immunosuppression; M, male; MSC, mesenchymal stem cells; PCD, primary ciliary dyskinesia; Pred, prednisone; Re-dose MSC, prior MSC infusion in phase Ia trial; Tac, tacrolimus; Tx, transplant; α-1 AT, alpha-1 antitrypsin deficiency.

Withdrew from study after MSC infusion.

**Table 2.** Cell count, viability, and sterility of infused MSCs in 13 subjects.

Group	Patient	Weight, kg	Prescribed cell dose, million/kg	Total number of cells infused	Pre-infusion viability, %	Gram stain	Bacterial/fungal culture
1	1	99.8	1.0	7.50E+07	76.5	NOS	Negative
	2	94.8	1.0	7.51E+07	79.5	NOS	Negative
	3	90.8	1.0	7.49E+07	72.1	NOS	Negative
2	4	87.5	0.5	3.75E+07	72.5	NOS	Negative
	5	66.9	0.5	2.49E+07	75.3	NOS	Negative
	6	74.9	0.5	2.50E+07	82.3	NOS	Negative
	7	86.9	0.5	3.75E+07	77.9	NOS	Negative
	8	68.8	0.5	2.50E+07	80.7	NOS	Negative
3	9	95.4	1.0	7.50E+07	88.2	NOS	Negative
	10	70.4	1.0	5.00E+07	86.1	NOS	Negative
	11	77.6	1.0	7.50E+07	89.7	NOS	Negative
	12	92.1	1.0	7.50E+07	76.6	NOS	Negative
	13	70.5	1.0	5.00E+07	77.4	NOS	Negative
Total patient population							
	Mean	82.8	0.8	5.38E+07	79.6		
	SD	11.6	0.3	2.19E+07	5.6		
	Median	86.9	1.0	5.00E+07	77.9		
	Minimum	66.9	0.5	2.49E+07	72.1		
	Maximum	99.8	1.0	7.51E+07	89.7		

Abbreviations: MSC, mesenchymal stem cells; NOS, no organism seen; SD, standard deviation.

### Subject Monitoring

Safety was evaluated by monitoring patients for their capacity to tolerate intravenous infusion without the development of toxicities and adverse reactions. Feasibility was evaluated by assessing the ability to recruit patients and by determination of logistical issues associated with product preparation and delivery to the clinical unit. Laboratory testing, including complete blood count and liver and renal function tests, was performed for all subjects prior to MSC infusion (day −7 to 1) and on days 1 and 7 post-infusion. Pulmonary function testing (PFT), including FVC and FEV1, was conducted on days −7 to −1 before infusion, on infusion day (day 0), and days 1, 7, 30, 90, 180, 270, and 365 post-infusion. Wherever possible, historical spirometry values were obtained at days −365 and −180 ± 30 prior to infusion. A single patient, transplanted elsewhere, did not have historical PFT within the pre-treatment time frame parameters (−365 and −180 days). Baseline best historical FEV1 was obtained by averaging the best two historical post-transplant FEV1 values prior to participation in the study, measured at least 3 weeks apart. All patients had routine post-transplant monitoring every 3 months and when clinically indicated. Whole blood was collected from subjects on days 0, 1, and 7 for biomarker evaluation.

### MSC Manufacturing

BMSC manufacturing was performed at the Human Cellular Therapy Laboratory (HCTL) at Mayo Clinic in Jacksonville, Florida. Bone marrow was obtained from a healthy donor who underwent a comprehensive medical examination and completed an institutionally approved donor history questionnaire. Infectious disease marker testing HIV-1 and 2 Antibodies, HIV Nucleic Acid Testing (NAT), HTLV I and II Antibodies, Syphilis Screen, Hepatitis B Surface Antigen, Hepatitis B Core Antibody, HBV NAT, HCV Antibody, HCV NAT, *Trypanosoma cruzi* Antibody, West Nile Virus NAT, Zika ELISA, Zika PCR was performed by a Clinical Laboratory Improvement Amendments (CLIA)-approved Laboratory. After a medical evaluation of the donor, review of infectious disease testing results, and donor history questionnaire, donor eligibility determination was performed by the HCTL Medical Director. After informed consent was obtained, and following bone marrow aspiration, allogeneic BMSCs from this single healthy donor were created by expanding the adherent fraction of fresh bone marrow aspirate using the Quantum Cell Expansion System (Terumo BCT, Lakewood, CO).^24, 25^ MSCs were cultured in α-minimum essential medium (Thermo Fisher Scientific, Waltham, MA), supplemented with 5% pooled Human Platelet Lysate (Sexton Biotechnologies, Indianapolis, IN), and 1× GlutaMAX (Thermo Fisher Scientific). Final cell products were cryopreserved at 2.5 × 10^6^ MSCs/mL in 20 mL CryoStor CS10 (10% dimethyl sulfoxide) (STEMCELL Technologies, Vancouver, BC) and stored in vapor phase liquid nitrogen at less than −150°C. Quality control testing was performed on the final cryopreserved cell product prior to release.

### MSC Preparation and Infusion

On the day of infusion, final cryopreserved MSC products were thawed and diluted 5-fold with Plasma-Lyte A (Baxter, Deerfield, IL) to yield a concentration of 2.0% DMSO and 0.5-1 million MSC/kg which were infused intravenously as outlined in Table 2. An aliquot of the final formulated product was reserved for cell count, viability testing, Gram staining, and bacterial/fungal culture evaluation.

Infusion of MSCs was performed in the clinical apheresis unit. MSCs were infused at a rate of 3-5 mL/minute for the first 15 minutes and subsequently adjusted based on tolerability. Patients were monitored for the occurrence of any adverse reactions, and infusion toxicity was evaluated by continuously monitoring the subject’s vital signs, before, during, and up to two hours after MSC infusion (Table 3). Five patients in group 2 received 0.5 × 10^6^ MSC/kg whereas 1 × 10^6^ MSC/kg was administered to the three subjects in group 1, and the five subjects in group 3 (Tables 1, 2). Doses were chosen based on the maximal effect of dosing 1 × 10^6^ cells/kg as opposed to 4 × 10^6^ cells/kg in a prior phase Ia study. A lower dose of 0.5 × 10^6^ was chosen to measure the effectiveness of an even lower dose. A paradoxical inverse dose response has previously been reported when administering MSCs to patients with cardiomyopathy.^26^

**Table 3.** Clinical parameters on day of infusion.

Variables measured at the time of MSC infusion	Pre-infusion Mean ± SD	0.5 hours after infusion Mean ± SD	1 hour after infusion Mean ± SD	1.5 hours after infusion Mean ± SD	2 hours after infusion Mean ± SD
Heart rate, bpm	73 ± 6	72 ± 8	74 ± 9	73 ± 7	74 ± 6
BP mean, mmHg	95 ± 10	91 ± 8	91 ± 10	92 ± 10	91 ± 7
Respiratory rate, Brpm	18 ± 2	17 ± 2	17 ± 2	17 ± 2	17 ± 2
Temperature, °C	36.7 ± 0.2	36.7 ± 0.1	36.7 ± 0.2	36.7 ± 0.1	36.7 ± 0.2
Oxygen saturation, %	97 ± 2	97 ± 2	97 ± 1	97 ± 2	97 ± 0.1
Borg dyspnea index	0.04 ± 0.14	0.15 ± 0.55	0.08 ± 0.19	0.04 ± 0.14	0.04 ± 0.14

Abbreviations: bpm, beats per minute; BP mean, mean arterial blood pressure; Brpm, breaths per minute.

### Statistical Analysis

All variables are summarized as mean ± SD or median (range). Individual variables were compared over time using paired *t* test. A *P* value of .05 was considered significant. All analysis was completed using SAS version 9.4 (Cary, NC).

## Results

### MSC Product

An average of 5.38 × 10^7^ MSCs were intravenously infused for each subject. The viability of the cell product prior to infusion was determined as 79.6% ± 5.6% by flow cytometry 7AAD staining. Among the three groups, no significant differences in viability were observed. All post-thaw bacterial/fungal cultures were negative and “no organisms seen” was reported for all Gram Stain evaluations performed (Table 2).

### Tolerance of MSC Infusion

There was no detrimental change to vital signs after infusion. Compared to baseline values measured before infusion, heart rate, BP mean, respiratory rate, temperature, oxygen saturation, and Borg dyspnea index measured at 0.5-, 1-, 1.5-, and 2-hour post-infusion were not significantly different (Table 3).

### Clinical Events Following MSC Infusion

There were no major clinical events during, immediately after (within the first week), or up to 1 month after MSC infusion. Significant clinical events occurring within the first year of follow-up are included in Supplementary Table S1. Two patients died within the 12-month period of follow-up after MSC infusion. One developed progressive left ventricular failure and renal failure 10 months after MSC infusion and requested palliation (group 3). The other (group 1) developed acute on chronic respiratory failure following an aspiration event 4 months after MSC infusion and requested palliation. Two patients (groups 2 and 3) were diagnosed and treated for new squamous cell cancer (SCC). Both had a history of treatment for SCC at different sites prior to enrollment. One patient (group 2) suffered a focal seizure deemed likely secondary to an old cerebral infarction, which had occurred years before enrollment. One patient (group 3) was treated for a respiratory infection 45 days after MSC infusion and recovered without sequelae. Since we do not have a control population, it is impossible to define whether any of these events were related to MSC infusion.

### Pulmonary Function Testing

**Figure 1.**
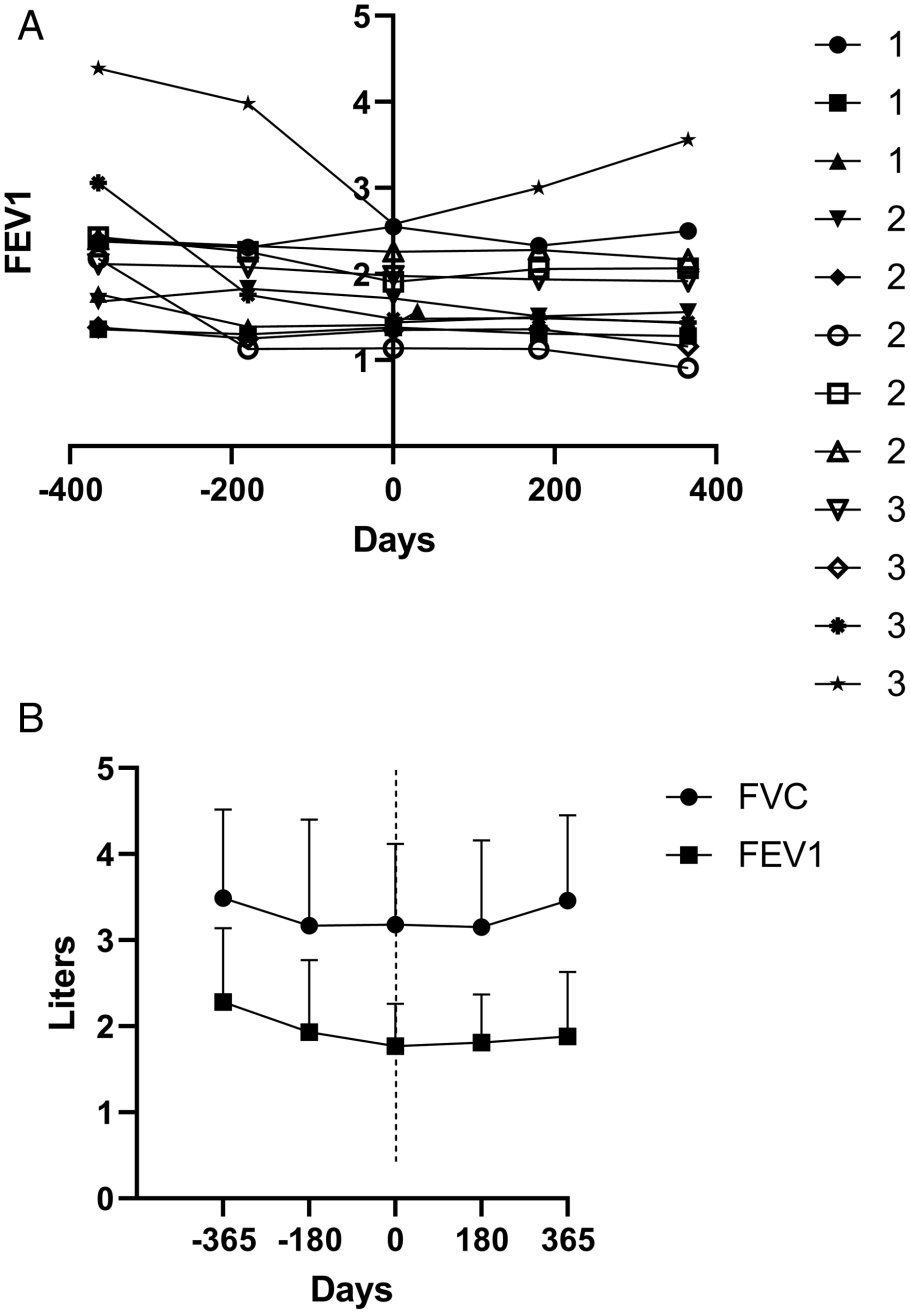
(a) Change in FEV1 before and after mesenchymal stem cell infusion on day 0. Groups 1 and 3: 1 million MSC/kg. Group 2: 0.5 million MSC/kg. (b) Mean ± SD change in FVC and FEV1 before and after MSC infusion for groups 1 and 2 and 3 combined. Compared to *t* = 0, *P* = .03, 0.04 for FVC and FEV1 at day −365; compared to *t* = 0, *P* = .84, 0.72 for FVC and FEV1 at day +365. Abbreviations: FEV1, forced expiratory volume in 1 second; FVC, forced vital capacity; MSC, mesenchymal stem cells.

Changes in pulmonary function at days −365, −180, and 0 prior to infusion of MSCs were compared to changes occurring on days 180 and 365 after MSC therapy and are presented in Table 4. Findings were not significantly different from those recorded in the previous phase Ia study.^22^ The dataset was incomplete at days 180 and 365 for one patient in group 1 and one patient in group 3 who died within the first year of follow-up. One patient in group 2 did not have historical PFT within the −365- and −180-day time frame before MSC infusion but had sufficient PFT to establish a diagnosis of obstructive CLAD at day 0. Overall, there was a significant decline in FVC (*P* = .03) and FEV1 (*P* = .04) from day −365 prior to therapy compared to the mean FVC and FEV1 measured immediately prior to MSC infusion on day 0. During the year following infusion, the mean FVC (*P* = .59, *P* = .53) and FEV1 (*P* = .84, *P* = .72) were not significantly changed on days 180 and 365 compared to days 0 (Table 4). Figure 1a shows individual changes in FEV1 for the 3 groups of patients according to whether they were re-dosed (group 1) with a dose of 1 × 10^6^ MSC/kg or received a first dose (group 3) at 1 × 10^6^ MSC/kg or (group 2) 0.5 × 10^6^ MSC/kg. Figure 1b shows the combined change in FVC and FEV1 before and after MSC infusion for the 3 groups. The dataset on day 365 did not include one patent from group 1 and one patient from group 3, who died during the year of follow-up. One patient in group 3 withdrew from the study as noted above. Stabilization in FEV1 at day 365 compared to day 0 indicates a change in pattern from the significant decline noted over the year preceding infusion. Patient 12 showed significant improvement in FVC and FEV1 on days 180 and 365 following MSC infusion.

**Table 4.** Changes in FVC and FEV1 before and after MSC infusion.

Dosing groups phase 1b	FVC pre-infusion day −365	FVC pre-infusion day −180	FVC pre-infusion day 0	FVC post-infusion day 180	FVC post-infusion day 365	FEV1 pre-infusion day −365	FEV1 pre-infusion day −180	FEV1 pre-infusion day 0	FEV1 post-infusion day 180	FEV1 post-infusion day 365
Group 1 Re-dose	4.41	4.01	4.44	3.7	4.49	2.38	2.31	2.55	2.33	2.5
	2.13	1.97	2.1	2	2.11	1.36	1.3	1.38	1.31	1.28
	3.24	2.87	2.89			1.77	1.39	1.41		
Group 2	3.41	3.53	3.63	2.93	3.06	1.68	1.83	1.72	1.51	1.56
			3.56	3.69	3.64			1.44	1.5	1.43
	2.64	1.55	1.56	1.55	1.85	2.18	1.13	1.14	1.13	0.91
	3.73	3.81	3.44	3.77	3.46	2.43	2.26	1.91	2.06	2.07
	4.11		3.84	3.86	3.88	2.4		2.26	2.28	2.17
Group 3	3.62	3.53	3.42	3.33	3.37	2.12	2.08	1.98	1.94	1.92
	1.74	1.53	1.65	1.59		1.38	1.25	1.35	1.36	
	4.07	3.42	3.68	3.67	3.56	3.06	1.76	1.48	1.49	1.44
	5.3	5.5	4.05	4.59	5.2	4.39	3.98	2.58	3	3.56
^a^	2.16	2.1	1.71			1.82	1.72	1.04		
Mean ± SD	3.49 ± 1.03^b^	3.17 ± 1.23	3.18 ± 0.94	3.15 ± 1.01	3.46 ± 0.99	2.28 ± 0.86^b^	1.93 ± 0.84	1.77 ± 0.49	1.81 ± 0.56	1.88 ± 0.75
Median (range)	3.62 (1.7-5.3)	3.48 (1.53-5.5)	3.5 (1.56-4.44)	3.67 (1.55-4.59)	3.51 (1.85-5.2)	2.18 (1.36-4.39)	1.8 (1.13-3.98)	1.6 (1.14-2.58)	1.51 (1.13-3)	1.74 (0.9-3.56)

Withdrew from study, spirometry not included in final analysis.

*P* < .05 compared to day 0, group 1: 1 × 10^6^ MSC/kg, group 2: 1 × 10^6^ MSC/kg, group 3: 0.5 × 10^6^ MSC/kg.

Abbreviations: FEV1, forced expiratory volume in 1 second; FVC, forced vital capacity; MSC, mesenchymal stem cells.

### Effect of MSC Therapy on Immune Effector Cells

**Figure 2.**
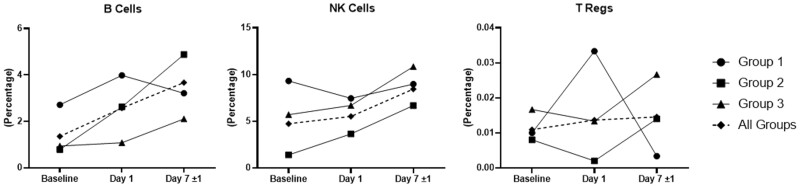
Circulating immune cells stratified by MSC dose groups at baseline, and days 1 and 7 ± 1 after infusion. Data for group 1 includes 3 subjects, data for group 2 include 5 subjects, and data for group 3 include 3 out of the 5 subjects. Abbreviation: MSC, mesenchymal stem cells.

We evaluated immune effector cells (NK, B, and Tregs) in the blood samples from 11 out of the 13 study subjects (Fig. 2). For group 2 and 3 subjects, there was an overall increase in the percentage of B and NK cells at day 7 ± 1 when compared to baseline. Compared to baseline, the percentage of Tregs decreased on day 1 and then increased on day 7 ± 1 for groups 2 and 3. Analysis of group 1 data did not produce a readily identifiable trend for the cell types evaluated. Overall, looking at the data for all subjects in aggregate (dashed lines), there was an increase in the number of B cells, NK cells, and Tregs from baseline to day 7 ± 1, with the increase in B-cell number being the most pronounced.

## Discussion

MSC are trapped in the vasculature of the lung after intravenous infusion. This “pulmonary first-pass effect” poses problems for administration to sites of injury other than the lung but may be particularly advantageous when the lung is the target site.^17^ We have previously shown that MSCs are indeed trapped in lung vasculature after intravenous infusion.^22^ Furthermore, the inflamed lung may exert a chemoattractant effect on MSCs. The debate as to whether MSCs primarily exert their influence through direct contact or by altering the micro-environment continues. This effect may include soluble factor release, including growth factors or cytokines, or via the release of lipid microvesicles.^27, 28^ Studies have demonstrated MSC response to chemokines, such as SDF-1 and MCF-3^29, 30^ and inflammatory cytokines tumor necrosis factor α, IL-1β, and IL-1α are required to induce immunosuppression by MSCs through the concerted action of chemokines and NO.^31^ Therefore, MSCs may be particularly suited to exerting their effect on inflamed lungs, such as with chronic lung allograft dysfunction. In a previous study, our group demonstrated a decrease in pro-inflammatory cytokines IL-6 and IL-8 in some patients and an increase in tolerogenic cytokine IL-4. Additionally, patients receiving lower-dose MSCs had also demonstrated an increase in epidermal growth factor in the serum, which may have a favorable effect on MSC-induced wound healing and tissue regeneration.^32^ In our previous phase Ia trial,^22^ we had demonstrated a more profound effect on biomarkers at the lowest dose (1 × 10^6^ MSC/kg). This dose was repeated for groups 1 and 3 in this study. An even lower dose (0.5 × 10^6^ MSC/kg) was chosen for group 2. MSCs used for ischemic heart failure have similarly demonstrated the greatest improvement in left ventricular function at low but not at high dose,^33^ and MSCs used to treat GVHD have shown no difference in safety profile or effect at low (2 × 10^6^ MSC/kg) vs high (8 × 10^6^ MSC/kg) dose.^34^

This clinical trial demonstrates that BMSCs (HCTL, Mayo Clinic Florida) were well tolerated with no demonstrable short-term adverse effects. MSCs at doses of 1 × 10^6^ and 0.5 × 10^6^ cells/kg may be safely administered to lung transplant recipients. Several studies have demonstrated safety when administering to patients with other lung diseases, such as chronic obstructive pulmonary disease, GVHD, and adult respiratory distress syndrome.^35–37^ Doses of 1 × 10^6^, 2 × 10^6^, and 4 × 10^6^ cells/kg were well tolerated in a prior study of lung transplant recipients at our institution.^22^ Chambers et al had previously shown that MSC from 5 different donors was safely administered to a more heterogeneous population of lung transplant recipients.^38^ Clinical events which occurred during the 12-month follow-up period were not unexpected in this elderly population of lung transplant recipients who had survived an average of 7.8 ± 3.4 years since transplant at the time MSCs were infused. There was no further decline in lung function over 12 months after MSCs were administered, suggesting stabilization in lung function after MSC infusion. All but patient 12 (in group 3) had an obstructive CLAD diagnosis established for more than 6 months before they were enrolled. Patient 12 had been diagnosed with recent-onset moderate obstructive CLAD less than 3 months before being infused. This patient also showed the most dramatic response, with spirometry values significantly improving at days 180 and 365 compared to day 0. In this small study, demographics were skewed toward a diagnosis of idiopathic pulmonary fibrosis (*n* = 8), elderly, and male (11 of 13) patients.

This study has limitations. As a phase I trial, the population was too small to make conclusions regarding efficacy. All but 1 patient had an established diagnosis of CLAD for more than 6 months before enrollment. MSC at 1 × 10^6^ or 0.5 × 10^6^ cells per kg can be safely administered to lung transplant recipients. Preliminary findings suggest there may be a stabilizing effect on progressive lung function decline in patients with obstructive CLAD. Except for 1 patient, these infusions were administered to patients with a well-established CLAD diagnosis of >6 months since onset. We believe there would be value in measuring the effect of MSCs on lung transplant patients with mild or early-onset CLAD in a phase II study. MSCs may provide greater impact before the pathological changes of CLAD are set, such as fibrotic changes of the restrictive phenotype, or the irreversible changes of bronchiolitis in the obstructive phenotype. We did not see an obvious paradoxical inverse dose response at the lowest dose. We propose using a dose of 1 × 10^6^ cells/kg in a possible phase II trial, as a similar stabilizing effect on lung function was observed in our previous phase I study at this dose.^22^

## Conclusion

BMSCs may be safely administered to lung transplant recipients with moderate to severe, treatment-refractory obstructive CLAD (BOS). Preliminary results suggest that larger, randomized prospective studies are warranted.

## Supplementary Material

szac051_suppl_Supplementary_Table_S1

## Data Availability

The data that support the findings of this study are available from the corresponding author upon reasonable request.

## References

[1] Weill D , et al. A consensus document for the selection of lung transplant candidates: 2014—an update from the Pulmonary Transplantation Council of the International Society for Heart and Lung Transplantation. J Heart Lung Transplant. 2015;34(1):1-15. 25085497 10.1016/j.healun.2014.06.014

[2] Kulkarni HS , et al. Bronchiolitis obliterans syndrome-free survival after lung transplantation: an International Society for Heart and Lung Transplantation Thoracic Transplant Registry analysis. J Heart Lung Transplant. 2019;38(1):5-16. 30391193 10.1016/j.healun.2018.09.016 PMC6431291

[3] Bankier AA , et al. Bronchiolitis obliterans syndrome in heart-lung transplant recipients: diagnosis with expiratory CT. Radiology. 2001;218(2):533-539. 11161175 10.1148/radiology.218.2.r01fe09533

[4] Sato M , et al. Time-dependent changes in the risk of death in pure bronchiolitis obliterans syndrome (BOS). J Heart Lung Transplant. 2013;32(5):484-491. 23433813 10.1016/j.healun.2013.01.1054

[5] Sato M , et al. Restrictive allograft syndrome (RAS): a novel form of chronic lung allograft dysfunction. J Heart Lung Transplant. 2011;30(7):735-742. 21419659 10.1016/j.healun.2011.01.712

[6] Christie JD , et al. Impact of primary graft failure on outcomes following lung transplantation. Chest. 2005;127(1):161-165. 15653978 10.1378/chest.127.1.161

[7] Burguete SR , et al. Lung transplant infection. Respirology. 2013;18(1):22-38. 22591266 10.1111/j.1440-1843.2012.02196.x PMC7192226

[8] Martinu T , Chen DF, Palmer SM. Acute rejection and humoral sensitization in lung transplant recipients. Proc Am Thorac Soc. 2009;6(1):54-65. 19131531 10.1513/pats.200808-080GO PMC2626504

[9] Witt CA , et al. Acute antibody-mediated rejection after lung transplantation. J Heart Lung Transplant. 2013;32(10):1034-1040. 23953920 10.1016/j.healun.2013.07.004 PMC3822761

[10] Corris PA , et al. A randomised controlled trial of azithromycin therapy in bronchiolitis obliterans syndrome (BOS) post lung transplantation. Thorax. 2015;70(5):442-450. 25714615 10.1136/thoraxjnl-2014-205998 PMC4413845

[11] Glanville AR. Montelukast for chronic lung allograft dysfunction: not quite the “Full Monty”. J Heart Lung Transplant. 2019;38(5):528-529. 30765304 10.1016/j.healun.2019.01.1312

[12] Meyer KC , et al. An international ISHLT/ATS/ERS clinical practice guideline: diagnosis and management of bronchiolitis obliterans syndrome. Eur Respir J. 2014;44(6):1479-1503. 25359357 10.1183/09031936.00107514

[13] Belperio JA , et al. Chronic lung allograft rejection: mechanisms and therapy. Proc Am Thorac Soc. 2009;6(1):108-121. 19131536 10.1513/pats.200807-073GO

[14] Davis RD Jr , et al. Improved lung allograft function after fundoplication in patients with gastroesophageal reflux disease undergoing lung transplantation. J Thorac Cardiovasc Surg. 2003;125(3):533-542. 12658195 10.1067/mtc.2003.166

[15] Yusen RD , et al. The registry of the International Society for Heart and Lung Transplantation: thirty-first adult lung and heart-lung transplant report—2014; focus theme: retransplantation. J Heart Lung Transplant. 2014;33(10):1009-1024. 25242125 10.1016/j.healun.2014.08.004

[16] Novick RJ , et al. Pulmonary retransplantation: predictors of graft function and survival in 230 patients. Pulmonary Retransplant Registry. Ann Thorac Surg. 1998;65(1):227-234. 9456123 10.1016/s0003-4975(97)01191-0

[17] Fischer UM , et al. Pulmonary passage is a major obstacle for intravenous stem cell delivery: the pulmonary first-pass effect. Stem Cells Dev. 2009;18(5):683-692. 19099374 10.1089/scd.2008.0253 PMC3190292

[18] Ortiz LA , et al. Mesenchymal stem cell engraftment in lung is enhanced in response to bleomycin exposure and ameliorates its fibrotic effects. Proc Natl Acad Sci USA. 2003;100(14):8407-8411. 12815096 10.1073/pnas.1432929100 PMC166242

[19] Kode JA , et al. Mesenchymal stem cells: immunobiology and role in immunomodulation and tissue regeneration. Cytotherapy. 2009;11(4):377-391. 19568970 10.1080/14653240903080367

[20] Spaggiari GM , et al. Mesenchymal stem cells inhibit natural killer-cell proliferation, cytotoxicity, and cytokine production: role of indoleamine 2,3-dioxygenase and prostaglandin E2. Blood. 2008;111(3):1327-1333. 17951526 10.1182/blood-2007-02-074997

[21] Weng JY , et al. Mesenchymal stem cell as salvage treatment for refractory chronic GVHD. Bone Marrow Transplant. 2010;45(12):1732-1740. 20818445 10.1038/bmt.2010.195 PMC3035976

[22] Keller CA , et al. Feasibility, safety, and tolerance of mesenchymal stem cell therapy for obstructive chronic lung allograft dysfunction. Stem Cells Transl Med. 2018;7(2):161-167. 29322685 10.1002/sctm.17-0198 PMC5788872

[23] Chambers DC , et al. Mesenchymal stromal cell therapy for chronic lung allograft dysfunction: results of a first-in-man study. Stem Cells Transl Med. 2017;6(4):1152-1157. 28186707 10.1002/sctm.16-0372 PMC5442848

[24] Hanley PJ , et al. Efficient manufacturing of therapeutic mesenchymal stromal cells with the use of the Quantum Cell Expansion System. Cytotherapy. 2014;16(8):1048-1058. 24726657 10.1016/j.jcyt.2014.01.417 PMC4087082

[25] Russell AL , Lefavor RC, Zubair AC. Characterization and cost-benefit analysis of automated bioreactor-expanded mesenchymal stem cells for clinical applications. Transfusion. 2018;58(10):2374-2382. 30203447 10.1111/trf.14805

[26] Hare JM , et al. Comparison of allogeneic vs autologous bone marrow-derived mesenchymal stem cells delivered by transendocardial injection in patients with ischemic cardiomyopathy: the POSEIDON randomized trial. JAMA. 2012;308(22):2369-2379. 23117550 10.1001/jama.2012.25321 PMC4762261

[27] Baglio SR , Pegtel DM, Baldini N. Mesenchymal stem cell secreted vesicles provide novel opportunities in (stem) cell-free therapy. Front Physiol. 2012;3:359. 22973239 10.3389/fphys.2012.00359 PMC3434369

[28] Rojas M , et al. Bone marrow-derived mesenchymal stem cells in repair of the injured lung. Am J Respir Cell Mol Biol. 2005;33(2):145-152. 15891110 10.1165/rcmb.2004-0330OC PMC2715309

[29] Kitaori T , et al. Stromal cell-derived factor 1/CXCR4 signaling is critical for the recruitment of mesenchymal stem cells to the fracture site during skeletal repair in a mouse model. Arthritis Rheum. 2009;60(3):813-823. 19248097 10.1002/art.24330

[30] Schenk S , et al. Monocyte chemotactic protein-3 is a myocardial mesenchymal stem cell homing factor. Stem Cells. 2007;25(1):245-251. 17053210 10.1634/stemcells.2006-0293

[31] Ren G , et al. Mesenchymal stem cell-mediated immunosuppression occurs via concerted action of chemokines and nitric oxide. Cell Stem Cell. 2008;2(2):141-150. 18371435 10.1016/j.stem.2007.11.014

[32] Khalili S , et al. Mesenchymal stromal cells improve salivary function and reduce lymphocytic infiltrates in mice with Sjogren’s-like disease. PLoS One. 2012;7(6):e38615. 22685592 10.1371/journal.pone.0038615 PMC3369846

[33] Perin EC , et al. A phase II dose-escalation study of allogeneic mesenchymal precursor cells in patients with ischemic or nonischemic heart failure. Circ Res. 2015;117(6):576-584. 26148930 10.1161/CIRCRESAHA.115.306332

[34] Kebriaei P , et al. Adult human mesenchymal stem cells added to corticosteroid therapy for the treatment of acute graft-versus-host disease. Biol Blood Marrow Transplant. 2009;15(7):804-811. 19539211 10.1016/j.bbmt.2008.03.012

[35] Ringden O , et al. Mesenchymal stem cells for treatment of therapy-resistant graft-versus-host disease. Transplantation. 2006;81(10):1390-1397. 16732175 10.1097/01.tp.0000214462.63943.14

[36] Weiss DJ , et al. A placebo-controlled, randomized trial of mesenchymal stem cells in COPD. Chest. 2013;143(6):1590-1598. 23172272 10.1378/chest.12-2094 PMC4694112

[37] Wilson J , et al. Mesenchymal stem (stromal) cells for treatment of acute respiratory distress syndrome—authors’ reply. Lancet Respir Med. 2015;3(4):e12-e13. 10.1016/S2213-2600(15)00040-5 25890655

[38] Chambers DC , et al. The International Thoracic Organ Transplant Registry of the International Society for Heart and Lung Transplantation: thirty-sixth adult lung and heart-lung transplantation Report-2019; Focus theme: donor and recipient size match. J Heart Lung Transplant. 2019;38(10):1042-1055. 31548030 10.1016/j.healun.2019.08.001 PMC6816340

