# Supplementary material for: Feasibility and Safety of Low-Dose Mesenchymal Stem Cell Infusion in Lung Transplant Recipients
Source: Stem Cells Transl Med. 2022 Jul 26;11(9):891–9. doi: 10.1093/stcltm/szac051 (PMC9492292; doi:10.1093/stcltm/szac051)
Supplement: szac051_suppl_Supplementary_Table_S1 [file szac051_suppl_supplementary_table_s1.docx]

**TITLE PAGE**

**Feasibility and Safety of Low Dose Mesenchymal Stem Cell Infusion in Lung Transplant Recipients**

**Running head:** MSC after Lung Transplantation

**Authors:**

1. Erasmus DB: conception and design, provision of study patients, collection of data, manuscript writing; ORCID ID: 0000-0001-8851-7466
2. Durand N: collection of data, data analysis and manuscript writing
3. Alvarez FA: collection and assembly of data, provision of study patients
4. Narula T: collection and assembly of data, provision of study patients
5. Hodge DO: Data analysis and interpretation
6. Zubair AC: Conception and design, data analysis and interpretation, final approval of manuscript; ORCID ID: 0000-0003-4827-4740

**Name of Institution:**

Mayo Clinic in Florida

**Correspondence:**

Corresponding author: David Brett Erasmus

Address: Vanderbilt University Medical Center, 1161 21^st^ Avenue South, Room T-1218, Nashville TN 37232

Tel: 615-322-1711

Email: [David.erasmus@vumc.org](mailto:David.erasmus@vumc.org)

**Disclaimers:** none of the authors have any disclaimers

INTRODUCTION

Lung transplantation offers prospects for better longevity and quality of life for a variety of end-stage lung diseases unresponsive to medical or surgical interventions [1]. Despite improvements over the last decade, chronic lung allograft failure (CLAD) still limits long-term survival. In a retrospective cohort study of primary lung transplant recipients (1994-2011) reported to the International Society of Heart and Lung Transplantation Thoracic Transplant Registry, 79,896 person-years of follow-up showed that median BOS (obstructive CLAD) free survival to be 3.16 (95% CI, 2.99-3.3) and 3.58 (95% CI, 3.53-3.72) years for single vs. bilateral lung transplant recipients respectively. Almost 90% of single and bilateral lung transplant recipients achieved the composite outcome of BOS (obstructive CLAD) or death 10 years after transplantation [2]. Chronic rejection remains the most important factor limiting survival after lung transplantation. Several clinical phenotypes for CLAD have been identified, predominantly obstructive (bronchiolitis obliterans syndrome) or restrictive (restrictive allograft syndrome) [3-5]. All phenotypes are associated with a sustained decline in airflow, measured most reliably by a decline in forced expiratory volume in 1 second (FEV1) compared to baseline (average of 2 best FEV1 values post-transplant, measured at least 3 weeks apart), in the absence of acute rejection, airway stenosis or active infection. Several factors may predispose patients to develop CLAD. Repeated bacterial, fungal, or viral infection, episodes of acute rejection (both cell-mediated and humoral), gastroesophageal reflux with aspiration, vascular injury, and others have been implicated [6-9]. Medical therapy with azithromycin [10] or montelukast [11], enhanced immunosuppression [12, 13], extracorporeal photopheresis, or medical and surgical intervention for GERD [14] may stabilize some patients, but in others progressive decline is relentless. Re-transplantation as a last resort carries a worse prognosis than primary lung transplantation, and few patients qualify for re-transplantation [15, 16]. Better interventions are required if lung transplantation is to no longer lag other solid organs for long-term survival. The development of novel and well-tolerated therapies for CLAD remains a high priority.

Mesenchymal stem cells (MSCs) are preferentially trapped in the lung after intravenous infusion, particularly in a setting of acute inflammation [17, 18]. They are known to modulate the cellular immune system by suppressing effector T cells. They shift T-helper (Th) 1 to a Th2 immune response, thereby shifting immune responses toward an anti-inflammatory and tolerogenic phenotype [19, 20]. They also exert an effect on B cells, thereby reducing antibody production [20]. Autologous and allogeneic MSCs have been evaluated in the treatment of other inflammatory lung conditions such as graft versus host disease (GVHD) [21]. In a prior phase 1 study at our institution (referred to in this study as phase 1a), we administered a single dose of MSCs to 9 patients, divided into 3 groups according to dose (4x10^6^, 2x10^6,^ and 1x10^6^ cells/kg), with established moderate CLAD (6.6 ± 3.1 years post-transplant). All patients had an obstructive phenotype. Clinically, there was no discernible difference in gas exchange at 1 year and renal function was unaffected. Spirometry suggested a pattern of stabilization in FEV1 and forced vital capacity (FVC) over the year following infusion. The most beneficial biological effect of an increase in tolerance inducing Th2 cytokines and reduction in Th1 pro-inflammatory cytokines (Il-1-α, IL-6, IL-8) and Chemokines (MIP-1α, MIP-1β) appeared to occur in the group receiving the lowest dose of MSCs [22]. Others have also demonstrated that intravenous administration of bone-marrow-derived allogeneic MSC is well tolerated in lung transplant recipients with moderate to severe CLAD and may provide stabilization to lung function [23].

In this study, (referred to as phase 1b), we measured lung function and clinical parameters in lung transplant recipients with established moderate to severe CLAD and an obstructive phenotype after a single low dose infusion of MSCs. We also re-dosed a small number of patients (n=3) who had received MSCs in the previous (phase 1a) study. All patients received a single dose intravenous infusion of bone-marrow-derived mesenchymal stem cells.

MATERIALS AND METHODS

Patient Population / Trial design

Study patients were recruited from the clinical practice of the lung transplant program at Mayo Clinic in Jacksonville, Florida. This study was conducted under IND15807 from the US Food and Drug Administration (FDA), approved by the Mayo Clinic Institutional Review Board (Protocol # 14-000025), and registered at ClinicalTrials.gov NCT02181712. Informed consent was obtained from all study participants.

Patients diagnosed with moderate to severe obstructive CLAD (table 1), refractory to standard interventions were enrolled in this phase 1 study. Thirteen patients received MSC infusion, but one withdrew before the 60-day follow-up, after being deemed suitable for re-transplantation. Twelve patients (11 male and 1 female) completed follow-up. All subjects were enrolled between 7/23/2018 and 9/18/2020 and were followed for twelve months. Three patients who had participated in the phase 1a study received a second dose of MSCs in this phase 1b study (Group 1). Demographic characteristics are summarized in table 1. Thirteen patients (11 male and 2 female) were recipients of either bilateral (n=10) or single (n=3) lung transplants. Underlying pre-transplant diagnoses included idiopathic pulmonary fibrosis (n=8), fibrotic NSIP (n=1), idiopathic bronchiectasis (n=1), primary ciliary dyskinesia (n=1), cystic fibrosis (n=1) and alpha-1 antitrypsin deficiency with severe emphysema (n=1). Patients were generally of advanced age (mean 64.4 ± 11.2 years). The day of infusion was considered Day 0. Baseline immunosuppression agents are included in table 1. All patients were immunosuppressed by a calcineurin inhibitor and prednisone. All but one patient in group 1 and one in group 2 were also on a cell cycle inhibitor (mycophenolate or azathioprine). Baseline immune function was measured as follows: mononuclear cells were isolated from peripheral blood collected at Baseline, Days 1, and 7 ±1 using Ficoll-Paque PREMIUM 1.077 (Cytiva Marlborough, MA). Isolated MNCs were evaluated for B cells (CD45+, CD19+), NK cells (CD45+, CD56+), and T Regs (CD4+, CD25+) (Abs from Beckton Dickinson, Franklin Lakes, NJ). Data were acquired using an Accuri TM C6 Cytometer and analyzed using FCS express. None of the patients had received a monoclonal antibody infusion or GCSF (granulocyte colony-stimulating factor) within the year preceding MSC infusion. Baseline PRA (pre-formed antibody) data is included in table 2. Two patients had detectable pre-formed antibodies, but none were donor-specific.

Subject Monitoring

Safety was evaluated by monitoring patients for their capacity to tolerate IV infusion without the development of toxicities and adverse reactions. Feasibility was evaluated by assessing the ability to recruit patients and by determination of logistical issues associated with product preparation and delivery to the clinical unit. Laboratory testing including complete blood count, and liver and renal function tests were performed for all subjects prior to MSC Infusion (Day -7 to 1) and on days 1 and 7 post-infusion. Pulmonary function tests (PFT) including forced vital capacity (FVC) and forced expiratory volume (FEV1) were conducted on Days -7 to -1 before infusion, on Infusion Day (Day 0), and Days 1, 7, 30, 90, 180, 270 and 365 post-infusion. Wherever possible, historical spirometry values were obtained at days -365 and -180 ± 30 days prior to infusion. A single patient, transplanted elsewhere, did not have historical pulmonary function testing within the pre-treatment time-frame parameters (-365 and -180 days). Baseline best historical FEV1 was obtained by averaging the best two historical post-transplant FEV1 values prior to participation in the study, measured at least 3 weeks apart. All patients had routine post-transplant monitoring every 3 months and when clinically indicated. Whole blood was collected from subjects on Days 0, 1, and 7 for biomarker evaluation.

MSC Manufacturing

Bone-marrow-derived MSC (BMSC) manufacturing was performed at the Human Cellular Therapy Laboratory (HCTL) at Mayo Clinic in Jacksonville, Florida. Bone marrow was obtained from a healthy donor who underwent a comprehensive medical examination and completed an institutionally approved Donor History Questionnaire. Infectious disease marker testing [HIV-1 and 2 Antibodies, HIV Nucleic Acid Testing (NAT), HTLV I & II Antibodies, Syphilis screen, Hepatitis B Surface Antigen, Hepatitis B Core Antibody, HBV NAT, HCV Antibody, HCV NAT, Trypanosoma cruzi Antibody, West Nile Virus NAT, Zika ELISA, Zika PCR] was performed by a Clinical Laboratory Improvement Amendments (CLIA)-approved Laboratory. After a medical evaluation of the donor, review of infectious disease testing results, and donor history questionnaire, donor eligibility determination was performed by the HCTL Medical Director. After informed consent was obtained, and following bone-marrow aspiration, allogeneic BMSCs from this single healthy donor were created by expanding the adherent fraction of fresh bone marrow aspirate using the Quantum® Cell Expansion System (Terumo BCT, Lakewood CO) [24, 25]. MSCs were cultured in Minimum Essential Medium α (Thermo Fisher Scientific, Waltham, MA), supplemented with 5 % pooled Human Platelet Lysate (Sexton Biotechnologies Indianapolis, IN), and 1X GlutaMAXTM (Thermo Fisher Scientific, Waltham, MA). Final cell products were cryopreserved at 2.5 x106 MSCs/mL in 20 mL CryoStor CS10 (10% dimethyl sulfoxide) (Stem Cell Technologies, Vancouver, BC) and stored in vapor-phase liquid nitrogen at less than -150oC. Quality Control testing was performed on the final cryopreserved cell product prior to release.

MSC Preparation & Infusion

On the day of infusion, final cryopreserved MSC products were thawed and diluted five-fold with Plasma-Lyte A (Baxter, Deerfield, IL) to yield a concentration of 2.0% DMSO and 0.5-1 million MSC/kg which were infused intravenously as outlined in Table 2. An aliquot of the final formulated product was reserved for cell count, viability testing, Gram staining, and bacterial/fungal culture evaluation.

Infusion of MSCs was performed in the clinical apheresis unit. MSCs were infused at a rate of 3-5 mL/minute for the first 15 minutes and subsequently adjusted based on tolerability. Patients were monitored for the occurrence of any adverse reactions, and infusion toxicity was evaluated by continuously monitoring the subject’s vital signs, before, during, and up to two hours after MSC infusion (Table 3). Five patients in Group 2 received 0.5x106 MSC/kg while 1x106 MSC/kg was administered to the three subjects in Group 1, and the five subjects in Group 3 (Tables1,2). Doses were chosen based on the maximal effect of dosing 1x10^6^ cells/kg as opposed to 4x10^6^ cells/kg in a prior phase 1a study. A lower dose of 0.5x10^6^ was chosen to measure the effectiveness of an even lower dose. A paradoxical inverse dose-response has previously been reported when administering MSCs to patients with cardiomyopathy [38].

Statistical analysis:

All variables are summarized as mean ± SD or median (range). Individual variables were compared over time using paired t-test. A p-value of 0.05 was considered significant. All analysis was completed using SAS version 9.4 (Cary, NC).

RESULTS

MSC Product

An average of 5.38x 10^7^ MSCs were intravenously infused for each subject. The viability of the cell product prior to infusion was determined as 79.6 % ± 5.6 % by flow cytometry 7AAD staining. Among the three groups, no significant differences in viability were observed. All post-thaw bacterial/fungal cultures were negative and ‘no organisms seen’ was reported for all Gram Stain evaluations performed (table 2).

Tolerance of MSC Infusion

There was no detrimental change to vital signs after infusion. Compared to baseline values measured before infusion, heart rate, BP mean, respiratory rate, temperature, oxygen saturation, and Borg dyspnea index measured at 0.5, 1-, 1.5- and 2 hours post-infusion were not significantly different (Table 3).

Clinical Events Following MSC infusion

There were no major clinical events during, immediately after (within the first week), or up to one month after MSC infusion. Significant clinical events occurring within the first year of follow-up are included in supplemental table S. Two patients died within the 12-month period of follow-up after MSC infusion. One developed progressive left ventricular failure and renal failure 10 months after MSC infusion and requested palliation (Group 3). The other (Group 1) developed acute on chronic respiratory failure following an aspiration event 4 months after MSC infusion and requested palliation. Two patients (Groups 2,3) were diagnosed and treated for new squamous cell cancer (SCC). Both had a history of treatment for SCC at different sites prior to enrollment. One patient (Group 2) suffered a focal seizure deemed likely secondary to an old cerebral infarction, which had occurred years before enrollment. One patient (Group 3) was treated for a respiratory infection 45 days after MSC infusion and recovered without sequelae. Since we do not have a control population, it is impossible to define whether any of these events were related to MSC infusion.

Pulmonary Function testing:

Changes in pulmonary function at days -365, -180, and 0 prior to infusion of MSCs were compared to changes occurring on days 180 and 365 after MSC therapy and are presented in table 4. Findings were not significantly different from those recorded in the previous phase 1a study [22]. The data set was incomplete at day 180 and day 365 for one patient in group 1 and one patient in group 3 who died within the first year of follow-up. One patient in group 2 did not have historical pulmonary function testing within the -365- and -180-day timeframe before MSC infusion but had sufficient pulmonary function testing to establish a diagnosis of obstructive CLAD at day 0. Overall, there was a significant decline in FVC (p=0.03) and FEV1 (p=0.04) from day -365 prior to therapy compared to the mean FVC and FEV1 measured immediately prior to MSC infusion on day 0. During the year following infusion, the mean FVC (p=0.59, p=0.53) and FEV1 (p=0.84, p=0.72) were not significantly changed on days 180 and 365 compared to days 0 (table 4). Figure 1a shows individual changes in FEV1 for the 3 groups of patients according to whether they were re-dosed (group 1) with a dose of 1x10^6^ MSC/kg or received a first dose (group 3) at 1x10^6^ MSC/kg or (group 2) 0.5x10^6^ MSC/kg. Figure 1b shows the combined change in FVC and FEV1 before and after MSC infusion for the 3 groups. The dataset on day 365 did not include one patent from group 1 and one patient from group 3, who died during the year of follow-up. One patient in group 3 withdrew from the study as noted above. Stabilization in FEV1 at day 365 compared to day 0 indicates a change in pattern from the significant decline noted over the year preceding infusion. Patient 12 showed significant improvement in FVC and FEV1 on days 180 and 365 following MSC infusion.

Effect of MSC Therapy on immune effector cells:

We evaluated immune effector cells (NK, B, and T Regs) in the blood samples from 11 out of the 13 study subjects (Figure 2). For Group 2 & 3 subjects, there was an overall increase in the percentage of B and NK cells at Day 7 ± 1 when compared to baseline. Compared to baseline, the percentage of T Regs decreased on day 1 and then increased on Day 7 ± 1 for groups 2 & 3. Analysis of Group 1 data did not produce a readily identifiable trend for the cell types evaluated. Overall, looking at the data for all subjects in aggregate (dashed lines), there was an increase in the quantity of B Cells, NK Cells, and T Regs from baseline to Day 7 ± 1, with the increase in B cell number being the most pronounced.

DISCUSSION

MSC are trapped in the vasculature of the lung after intravenous infusion. This “pulmonary first-pass effect” poses problems for administration to sites of injury other than the lung but may be particularly advantageous when the lung is the target site [17]. We have previously shown that MSCs are indeed trapped in lung vasculature after intravenous infusion [22]. Furthermore, the inflamed lung may exert a chemoattractant effect on MSCs. The debate as to whether MSCs primarily exert their influence through direct contact or by altering the micro-environment continues. This effect may include soluble factor release, including growth factors or cytokines, or via the release of lipid micro-vesicles [26, 27]. Studies have demonstrated MSC response to chemokines such as SDF-1 and MCF-3 [28, 29] and inflammatory cytokines tumor necrosis factor α, IL-1β, and IL-1α are required to induce immunosuppression by MSCs through the concerted action of chemokines and NO [30]. Therefore, MSCs may be particularly suited to exerting their effect on inflamed lungs, such as with chronic lung allograft dysfunction. In a previous study, our group demonstrated a decrease in proinflammatory cytokines IL-6 and IL-8 in some patients and an increase in tolerogenic cytokine IL-4. Additionally, patients receiving lower dose MSCs had also demonstrated an increase in epidermal growth factor in serum, which may have a favorable effect on MSC-induced wound healing and tissue regeneration [31]. In our previous phase 1a trial [22], we had demonstrated a more profound effect on biomarkers at the lowest dose (1x10^6^ MSC/kg). This dose was repeated for groups 1 and 3 in this study. An even lower dose (0.5x10^6^ MSC/kg) was chosen for Group 2. MSCs used for ischemic heart failure have similarly demonstrated the greatest improvement in left ventricular function at low but not at high dose [32], and MSCs used to treat GVHD have shown no difference in safety profile or effect at low (2x10^6^ MSC/kg) vs high (8x10^6^ MSC/kg) dose [33].

This clinical trial demonstrates that bone-marrow-derived MSCs (Human Cell Therapy Laboratory (HCTL), Mayo Clinic Florida) was well tolerated with no demonstrable short-term adverse effects. MSCs at doses of 1x10^6^ and 0.5x10^6^ cells/kg may be safely administered to lung transplant recipients. Several studies have demonstrated safety when administering to patients with other lung diseases such as chronic obstructive pulmonary disease, GVHD, and adult respiratory distress syndrome [34-36]. Doses of 1x10^6^, 2x10^6^, and 4x10^6^ cells/kg were well-tolerated in a prior study of lung transplant recipients at our institution [22]. Chambers et al had previously shown that MSC from 5 different donors was safely administered to a more heterogeneous population of lung transplant recipients [37]. Clinical events which occurred during the 12-month follow-up period were not unexpected in this elderly population of lung transplant recipients who had survived an average of 7.8 ± 3.4 years since transplant at the time MSCs were infused. There was no further decline in lung function over 12 months after MSCs were administered, suggesting stabilization in lung function after MSC infusion. All but patient 12 (in group 3) had an obstructive CLAD diagnosis established for more than 6 months before they were enrolled. Patient 12 had been diagnosed with recent-onset moderate obstructive CLAD less than 3 months before being infused. This patient also showed the most dramatic response, with spirometry values significantly improving at days 180 and 365 compared to day 0. In this small study, demographics were skewed towards a diagnosis of idiopathic pulmonary fibrosis (n=8), elderly, and male (11 of 13) patients.

This study has limitations. As a phase 1 trial, the population was too small to make conclusions regarding efficacy. All but 1 patient had an established diagnosis of CLAD for more than 6 months before enrollment. MSC at 1x10^6^ or 0.5x10^6^ Cells per kg can be safely administered to lung transplant recipients. Preliminary findings suggest there may be a stabilizing effect on progressive lung function decline in patients with obstructive CLAD. Except for 1 patient, these infusions were administered to patients with a well-established CLAD diagnosis of > 6 months since onset. We believe there would be value in measuring the effect of MSCs on lung transplant patients with mild or early-onset CLAD in a phase 2 study. MSCs may provide greater impact before the pathological changes of CLAD are set, such as fibrotic changes of the restrictive phenotype, or the irreversible changes of bronchiolitis in the obstructive phenotype. We did not see an obvious paradoxical inverse dose-response at the lowest dose. We propose using a dose of 1x10^6^ cells/kg in a possible phase 2 trial, as a similar stabilizing effect on lung function was observed in our previous phase 1 study at this dose [22].

CONCLUSION

Bone-marrow-derived mesenchymal stem cells may be safely administered to lung transplant recipients with moderate to severe, treatment-refractory obstructive CLAD (bronchiolitis obliterans syndrome). Preliminary results suggest that larger, randomized prospective studies are warranted.

1. Weill, D., et al., A consensus document for the selection of lung transplant candidates: 2014--an update from the Pulmonary Transplantation Council of the International Society for Heart and Lung Transplantation. J Heart Lung Transplant, 2015. 34(1): p. 1-15.

2. Kulkarni, H.S., et al., Bronchiolitis obliterans syndrome-free survival after lung transplantation: An International Society for Heart and Lung Transplantation Thoracic Transplant Registry analysis. J Heart Lung Transplant, 2019. 38(1): p. 5-16.

3. Bankier, A.A., et al., Bronchiolitis obliterans syndrome in heart-lung transplant recipients: diagnosis with expiratory CT. Radiology, 2001. 218(2): p. 533-9.

4. Sato, M., et al., Time-dependent changes in the risk of death in pure bronchiolitis obliterans syndrome (BOS). J Heart Lung Transplant, 2013. 32(5): p. 484-91.

5. Sato, M., et al., Restrictive allograft syndrome (RAS): a novel form of chronic lung allograft dysfunction. J Heart Lung Transplant, 2011. 30(7): p. 735-42.

6. Christie, J.D., et al., Impact of primary graft failure on outcomes following lung transplantation. Chest, 2005. 127(1): p. 161-5.

7. Burguete, S.R., et al., Lung transplant infection. Respirology, 2013. 18(1): p. 22-38.

8. Martinu, T., D.F. Chen, and S.M. Palmer, Acute rejection and humoral sensitization in lung transplant recipients. Proc Am Thorac Soc, 2009. 6(1): p. 54-65.

9. Witt, C.A., et al., Acute antibody-mediated rejection after lung transplantation. J Heart Lung Transplant, 2013. 32(10): p. 1034-40.

10. Corris, P.A., et al., A randomised controlled trial of azithromycin therapy in bronchiolitis obliterans syndrome (BOS) post lung transplantation. Thorax, 2015. 70(5): p. 442-50.

11. Glanville, A.R., Montelukast for chronic lung allograft dysfunction: Not quite the "Full Monty". J Heart Lung Transplant, 2019. 38(5): p. 528-529.

12. Meyer, K.C., et al., An international ISHLT/ATS/ERS clinical practice guideline: diagnosis and management of bronchiolitis obliterans syndrome. Eur Respir J, 2014. 44(6): p. 1479-503.

13. Belperio, J.A., et al., Chronic lung allograft rejection: mechanisms and therapy. Proc Am Thorac Soc, 2009. 6(1): p. 108-21.

14. Davis, R.D., Jr., et al., Improved lung allograft function after fundoplication in patients with gastroesophageal reflux disease undergoing lung transplantation. J Thorac Cardiovasc Surg, 2003. 125(3): p. 533-42.

15. Yusen, R.D., et al., The registry of the International Society for Heart and Lung Transplantation: thirty-first adult lung and heart-lung transplant report--2014; focus theme: retransplantation. J Heart Lung Transplant, 2014. 33(10): p. 1009-24.

16. Novick, R.J., et al., Pulmonary retransplantation: predictors of graft function and survival in 230 patients. Pulmonary Retransplant Registry. Ann Thorac Surg, 1998. 65(1): p. 227-34.

17. Fischer, U.M., et al., Pulmonary passage is a major obstacle for intravenous stem cell delivery: the pulmonary first-pass effect. Stem Cells Dev, 2009. 18(5): p. 683-92.

18. Ortiz, L.A., et al., Mesenchymal stem cell engraftment in lung is enhanced in response to bleomycin exposure and ameliorates its fibrotic effects. Proc Natl Acad Sci U S A, 2003. 100(14): p. 8407-11.

19. Kode, J.A., et al., Mesenchymal stem cells: immunobiology and role in immunomodulation and tissue regeneration. Cytotherapy, 2009. 11(4): p. 377-91.

20. Spaggiari, G.M., et al., Mesenchymal stem cells inhibit natural killer-cell proliferation, cytotoxicity, and cytokine production: role of indoleamine 2,3-dioxygenase and prostaglandin E2. Blood, 2008. 111(3): p. 1327-33.

21. Weng, J.Y., et al., Mesenchymal stem cell as salvage treatment for refractory chronic GVHD. Bone Marrow Transplant, 2010. 45(12): p. 1732-40.

22. Keller, C.A., et al., Feasibility, Safety, and Tolerance of Mesenchymal Stem Cell Therapy for Obstructive Chronic Lung Allograft Dysfunction. Stem Cells Transl Med, 2018. 7(2): p. 161-167.

23. Chambers, D.C., et al., Mesenchymal Stromal Cell Therapy for Chronic Lung Allograft Dysfunction: Results of a First-in-Man Study. Stem Cells Transl Med, 2017. 6(4): p. 1152-1157.

24. Hanley, P.J., et al., Efficient manufacturing of therapeutic mesenchymal stromal cells with the use of the Quantum Cell Expansion System. Cytotherapy, 2014. 16(8): p. 1048-58.

25. Russell, A.L., R.C. Lefavor, and A.C. Zubair, Characterization and cost-benefit analysis of automated bioreactor-expanded mesenchymal stem cells for clinical applications. Transfusion, 2018. 58(10): p. 2374-2382.

26. Baglio, S.R., D.M. Pegtel, and N. Baldini, Mesenchymal stem cell secreted vesicles provide novel opportunities in (stem) cell-free therapy. Front Physiol, 2012. 3: p. 359.

27. Rojas, M., et al., Bone marrow-derived mesenchymal stem cells in repair of the injured lung. Am J Respir Cell Mol Biol, 2005. 33(2): p. 145-52.

28. Kitaori, T., et al., Stromal cell-derived factor 1/CXCR4 signaling is critical for the recruitment of mesenchymal stem cells to the fracture site during skeletal repair in a mouse model. Arthritis Rheum, 2009. 60(3): p. 813-23.

29. Schenk, S., et al., Monocyte chemotactic protein-3 is a myocardial mesenchymal stem cell homing factor. Stem Cells, 2007. 25(1): p. 245-51.

30. Ren, G., et al., Mesenchymal stem cell-mediated immunosuppression occurs via concerted action of chemokines and nitric oxide. Cell Stem Cell, 2008. 2(2): p. 141-50.

31. Khalili, S., et al., Mesenchymal stromal cells improve salivary function and reduce lymphocytic infiltrates in mice with Sjogren's-like disease. PLoS One, 2012. 7(6): p. e38615.

32. Perin, E.C., et al., A Phase II Dose-Escalation Study of Allogeneic Mesenchymal Precursor Cells in Patients With Ischemic or Nonischemic Heart Failure. Circ Res, 2015. 117(6): p. 576-84.

33. Kebriaei, P., et al., Adult human mesenchymal stem cells added to corticosteroid therapy for the treatment of acute graft-versus-host disease. Biol Blood Marrow Transplant, 2009. 15(7): p. 804-11.

34. Ringden, O., et al., Mesenchymal stem cells for treatment of therapy-resistant graft-versus-host disease. Transplantation, 2006. 81(10): p. 1390-7.

35. Weiss, D.J., et al., A placebo-controlled, randomized trial of mesenchymal stem cells in COPD. Chest, 2013. 143(6): p. 1590-1598.

36. Wilson, J., et al., Mesenchymal stem (stromal) cells for treatment of acute respiratory distress syndrome - authors' reply. Lancet Respir Med, 2015. 3(4): p. e12-3.

37. Chambers, D.C., et al., The International Thoracic Organ Transplant Registry of the International Society for Heart and Lung Transplantation: Thirty-sixth adult lung and heart-lung transplantation Report-2019; Focus theme: Donor and recipient size match. J Heart Lung Transplant, 2019. 38(10): p. 1042-1055.

38. Hare, J.M., et al., Comparison of allogeneic vs autologous bone marrow-derived mesenchymal stem cells delivered by transendocardial injection in patients with ischemic cardiomyopathy: the POSEIDON randomized trial. JAMA, 2012. **308**(22): p. 2369-79.

The data that support the findings of this study are available from the corresponding author upon reasonable request.

Table 1: Demographic Characteristics of the 3 study groups

| Dosing | Age | | Sex | | Pre-Tx Diagnosis | IS | Type Tx | cPRA % | Re-dose MSC | FEV1 % decline at infusion | Interval from prior MSC dose (years) | Time: Tx to MSC infusion (years) |
| --- | --- | --- | --- | --- | --- | --- | --- | --- | --- | --- | --- | --- |
| Group 1  1x10^6^ MSC/kg | 74 | | M | | PCD | CyA, MMF, Pred | Bilateral | 0 | Yes | 49% | 3.05 | 10.3 |
|  | 73 | | M | | IPF | Tac, AZA, Pred | Single | 0 | Yes | 48% | 3.68 | 13.1 |
|  | 64 | | M | | IPF | Tac, MMF, Pred | Bilateral | 0 | Yes | 52% | 3.26 | 8.45 |
| Group 2  0.5x10^6^ MSC/kg | 71 | | M | | IPF | Tac,  Pred | Bilateral | 0 | No | 42% | NA | 6 |
|  | 36 | | M | | CF | Tac, MMF, Pred | Bilateral | 4 | No | 53% | NA | 6.5 |
|  | 65 | | F | | IPF | Tac, MMF, Pred | Bilateral | 0 | No | 49% | NA | 3.12 |
|  | 72 | | M | | IPF | Tac, MMF, Pred | Bilateral | 32 | No | 45% | NA | 5.02 |
|  | 70 | | M | | IPF | CyA,  Pred | Bilateral | 0 | No | 32% | NA | 8.75 |
| Group 3  1x10^6^ MSC/kg | 72 | | M | | BE | Tac, MMF, Pred | Bilateral | 0 | No | 46% | NA | 9.5 |
|  | 71 | | M | | IPF | Tac,  Pred | Single | 0 | No | 32% | NA | 12.75 |
|  | 62 | | M | | IPF | Tac, MMF, Pred | Bilateral | 0 | No | 48% | NA | 8.04 |
|  | 48 | | M | | α-1 AT | Tac, MMF, Pred | Bilateral | 0 | No | 38% | NA | 2.47 |
|  | 59** | | F | | f-NSIP | Tac, MMF, Pred | Single | 0 | No | 53% | NA | 4.79 |
| Mean ±SD | 64.4±11.2 | |  | |  |  |  |  |  | 45±7% |  | 7.6±3.4 |
|  | |  | | **Abbreviations:** F-female, M-male, MSC-mesenchymal stem cells, FEV1-forced expiratory volume in 1 second, Tx-transplant, Re-dose MSC-prior MSC infusion in phase 1a trial, PCD-primary ciliary dyskinesia, IPF-idiopathic pulmonary fibrosis, CF-cystic fibrosis, α-1 AT – alpha 1 antitrypsin deficiency, BE-bronchiectasis, f-NSIP-fibrosing NSIP, ** withdrew from study after MSC infusion, IS-Immunosuppression, CyA-Cyclosporine, Tac-Tacrolimus, Pred-Prednisone, AZA-Azathioprine | | | | | | | | |

Table 2


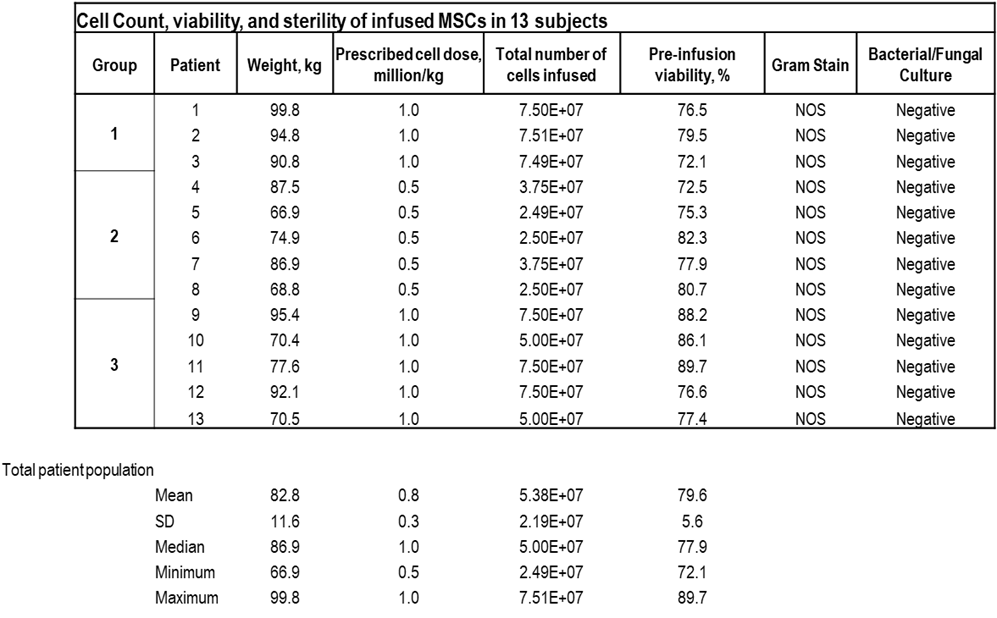


Abbreviations: MSC-mesenchymal stem cell; SD-standard deviation, NOS-no organism seen

Table 3: Clinical Parameters on Day of Infusion


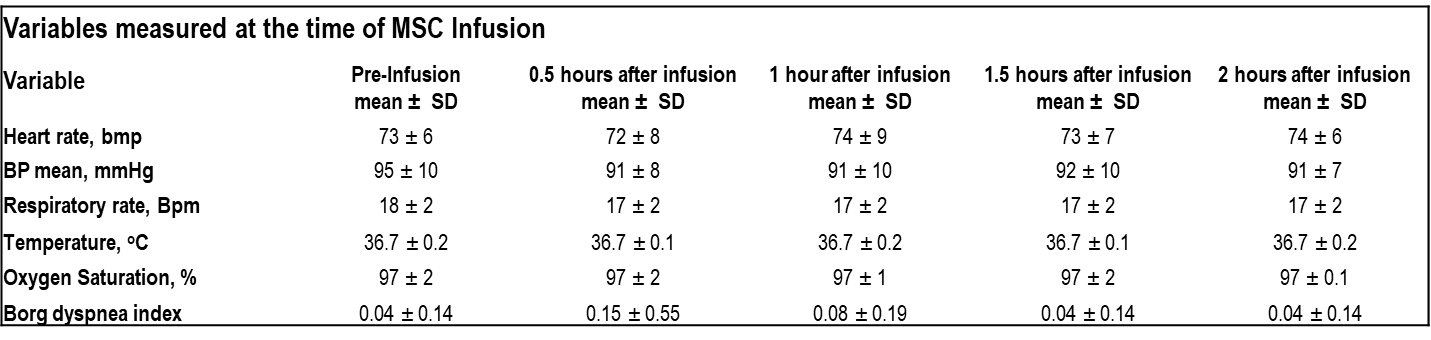


Abbreviations: BP mean- mean arterial blood pressure, bmp- beats per minute, Bpm- breaths per minute

Table 4: Changes in FVC and FEV1 before and after MSC infusion

| **Dosing**  **Groups**  **Phase 1b** | **FVC**  **Pre-infusion**  **Day -365** | **FVC**  **Pre-infusion**  **Day -180** | **FVC**  **Pre-infusion**  **Day 0** | **FVC**  **Post-infusion**  **Day 180** | **FVC**  **Post-infusion**  **Day 365** | **FEV1**  **Pre-infusion**  **Day -365** | **FEV1**  **Pre-infusion**  **Day -180** | **FEV1**  **Pre-infusion,**  **Day 0** | **FEV1 P**  **Post-infusion**  **Day 180** | **FEV1**  **Post-infusion**  **Day 365** |
| --- | --- | --- | --- | --- | --- | --- | --- | --- | --- | --- |
| Group 1  Re-dose | 4.41 | 4.01 | 4.44 | 3.7 | 4.49 | 2.38 | 2.31 | 2.55 | 2.33 | 2.5 |
|  | 2.13 | 1.97 | 2.1 | 2 | 2.11 | 1.36 | 1.3 | 1.38 | 1.31 | 1.28 |
|  | 3.24 | 2.87 | 2.89 |  |  | 1.77 | 1.39 | 1.41 |  |  |
| Group 2 | 3.41 | 3.53 | 3.63 | 2.93 | 3.06 | 1.68 | 1.83 | 1.72 | 1.51 | 1.56 |
|  |  |  | 3.56 | 3.69 | 3.64 |  |  | 1.44 | 1.5 | 1.43 |
|  | 2.64 | 1.55 | 1.56 | 1.55 | 1.85 | 2.18 | 1.13 | 1.14 | 1.13 | 0.91 |
|  | 3.73 | 3.81 | 3.44 | 3.77 | 3.46 | 2.43 | 2.26 | 1.91 | 2.06 | 2.07 |
|  | 4.11 |  | 3.84 | 3.86 | 3.88 | 2.4 |  | 2.26 | 2.28 | 2.17 |
| Group 3 | 3.62 | 3.53 | 3.42 | 3.33 | 3.37 | 2.12 | 2.08 | 1.98 | 1.94 | 1.92 |
|  | 1.74 | 1.53 | 1.65 | 1.59 |  | 1.38 | 1.25 | 1.35 | 1.36 |  |
|  | 4.07 | 3.42 | 3.68 | 3.67 | 3.56 | 3.06 | 1.76 | 1.48 | 1.49 | 1.44 |
|  | 5.3 | 5.5 | 4.05 | 4.59 | 5.2 | 4.39 | 3.98 | 2.58 | 3 | 3.56 |
| ** | 2.16 | 2.1 | 1.71 |  |  | 1.82 | 1.72 | 1.04 |  |  |
| Mean ± SD | 3.49±1.03* | 3.17±1.23 | 3.18±0.94 | 3.15±1.01 | 3.46±0.99 | 2.28±0.86* | 1.93±0.84 | 1.77±0.49 | 1.81±0.56 | 1.88±0.75 |
| Median (range) | 3.62  (1.7-5.3) | 3.48  (1.53-5.5) | 3.5  (1.56-4.44) | 3.67  (1.55-4.59) | 3.51  (1.85-5.2) | 2.18  (1.36-4.39) | 1.8  (1.13-3.98) | 1.6  (1.14-2.58) | 1.51  (1.13-3) | 1.74  (0.9-3.56) |
| Abbreviations: FVC – forced vital capacity, FEV1 – Forced expiratory volume in 1 second, MSC – mesenchymal stem cells, * - P < 0.05 compared to day 0, Group 1: 1x10^6^ MSC/kg, Group 2: 1x10^6^ MSC/kg, Group 3: 0.5x10^6^ MSC/kg; ** withdrew from study, spirometry not included in final analysis | | | | | | | | | | |

Table S: Clinical Events after MSC Infusion

| **Group** | **Subject** | **Significant clinical events over 12-month period following MSC** |
| --- | --- | --- |
| 1 | 1 | No significant events |
|  | 2 | Fell during 6 min walk, 11 months after MSC infusion |
|  | 3 | Developed infection/aspiration and progressive respiratory failure 4 months after MSC infusion. Palliative care consulted; died before 6-month follow-up. |
| 2 | 4 | Treated for respiratory infection 3 months after MSC infusion; symptoms improved after antibiotics |
|  | 5 | No significant events |
|  | 6 | No significant events |
|  | 7 | Focal seizure 1 year after MSC; prior (pre MSC) hemorrhagic infarction |
|  | 8 | New focal squamous cell skin cancer |
| 3 | 9 | New focal squamous cell skin cancer |
|  | 10 | Left ventricular failure/ renal failure 10 months after MSC infusion. Patient requested palliative care and died before 12-month follow-up |
|  | 11 | Acute respiratory infection 45 days after MSC infusion, symptoms resolved with antibiotics |
|  | 12 | No significant events |
|  | 13 | No significant events – withdrew from study before 60-day follow-up |
